# Genetics of zonal leaf chlorosis and genetic linkage to a major gene regulating skin anthocyanin production (*MdMYB1*) in the apple (*Malus* × *domestica*) cultivar Honeycrisp

**DOI:** 10.1371/journal.pone.0210611

**Published:** 2019-01-28

**Authors:** Nicholas P. Howard, John Tillman, Stijn Vanderzande, James J. Luby

**Affiliations:** 1 Department of Horticultural Science, University of Minnesota, St. Paul, Minnesota, United States of America; 2 Institut für Biologie und Umweltwissenschaften, Carl von Ossietzky Universität, Oldenburg, Germany; 3 Department of Horticulture and Landscape Architecture, Washington State University, Pullman, Washington, United States of America; USDA-ARS Southern Regional Research Center, UNITED STATES

## Abstract

‘Honeycrisp’ is a widely grown and acclaimed apple cultivar that is commonly used in breeding programs. It also has a well-documented tendency to develop the physiological disorder, zonal leaf chlorosis (ZLC). This disorder causes reduced photosynthetic capacity and is thought to be due to a problem with phloem loading, although the underlying genetics of the disorder have not previously been discerned. In order to understand the breeding implications of the disorder, six families with ‘Honeycrisp’ as a parent and one family with ‘Honeycrisp’ as both a maternal and paternal grandparent were evaluated for ZLC incidence over two years. One major quantitative trait locus (QTL) for ZLC incidence was identified on linkage group (LG) 9. A haplotype in ‘Honeycrisp’ that originated from grandparent ‘Duchess of Oldenburg’ was associated with increased ZLC incidence in offspring in both years and all families evaluated. The LG9 QTL was 5 to 10 cM from *MdMYB1*, which is a major gene regulating fruit skin anthocyanin production. ‘Honeycrisp’ is heterozygous for red fruit skin overcolor at *MdMYB1*. The ‘Honeycrisp’ haplotype at the LG9 QTL associated with increased ZLC is in linkage phase with the allele at *MdMYB1* associated with red color. Selection for the red allele from ‘Honeycrisp’ at *MdMYB1* will result in most offspring also inheriting the haplotype at the LG9 QTL associated with high ZLC. The occurrence of two copies of this haplotype was sub-lethal in seedlings of a family where both parents inherited both the red overcolor allele at MdMYB1 and the haplotype at the LG9 QTL associated with high ZLC. This is the first study to have identified a genetic component of ZLC with clear breeding implications.

## Introduction

‘Honeycrisp’ has become a popular apple (*Malus* × *domestica*) cultivar in U.S. markets [1] and an important parent in apple breeding programs worldwide. Its high acclaim has been largely due to its strong consumer preference [2], the uniquely ultra-crisp texture of its fruit [3] that is retained through extended cold storage [4], [5], [6] and its reported resistance to apple scab [7]. However, growers have been concerned about the tendency of ‘Honeycrisp’ trees to develop the leaf disorder termed zonal leaf chlorosis (ZLC) [5]. This disorder is characterized by yellowing of areas at the outer margins of leaves that cover more of the leaf surface as the season progresses. These chlorotic areas often turn necrotic by the end of the growing season leading to growers’ concerns about the unhealthy appearance of ‘Honeycrisp’ trees affected by ZLC [5], [8]. This concern, as well as interest in the molecular underpinnings of ZLC, has prompted several investigations into horticultural and physiological consequences of the disorder.

Severity of ZLC in ‘Honeycrisp’ trees has been associated with lower crop load in several studies [8], [9], [10], though this relationship was not observed in Fleck et al. [11]. Severity of ZLC in ‘Honeycrisp’ scions has also been observed to vary depending on which rootstock they have been grafted onto [12], [13].

‘Honeycrisp’ leaves affected by ZLC can have reduced photosynthetic capacity [11], [14]. An accumulation of many large starch granules has been observed in affected areas of leaves and was associated with damage to chloroplasts [10], [15]. These observations and similarities between ZLC and chlorosis disorders not related to nutrient deficiencies in other crops [16], [17], [18] have led investigators to believe that ZLC is due to a problem with phloem loading or transport from leaves to carbon sinks [10], [15].

To date, we are not aware that severe ZLC has been commonly reported in any other apple cultivar. However, populations of apple seedlings in the University of Minnesota (UMN) apple breeding program that have ‘Honeycrisp’ as a parent have been observed to segregate for ZLC. Considering the importance of ‘Honeycrisp’ as a breeding parent and grower concerns over ZLC, an understanding of the inheritance of ZLC could be useful in apple breeding. With that in mind, the focus of this study was to identify major QTL for ZLC and breeding implications of identified QTL.

## Materials and methods

### Plant material

Two populations were evaluated in this study (S1 and S2 Tables). The first population consisted of 409 genotypes from five families that share ‘Honeycrisp’ as a common parent. The other parents in this population were ‘Jonafree’ [19], ‘Monark’ (also known as the University of Arkansas selection AA-44) [20], ‘Pitmaston Pineapple’ (USDA PI 279323) and the UMN apple selections MN1702 (‘Fireside’ x ‘Frostbite’) and MN1764 (parentage unknown). These families have been previously described in McKay et al. [21] and were used to create the integrated genetic map described in Howard et al., 2017 [22] that is used in this study. Most genotypes in this population have two replicated trees, with some individuals in the ‘Honeycrisp’ x MN1764 family having three or four replicated trees. This population was evaluated from 2014 to 2016 at the UMN Horticultural Research Center in Chanhassen, Minnesota, USA. The trees from these families were budded onto ‘Budagovsky 9’ rootstock in 2008 (buds that failed or were damaged were rebudded, primarily in 2009 and 2010).

The second population consisted of 152 genotypes from a single family from a cross between ‘Minneiska’ (‘Honeycrisp’ x ‘Minnewashta’) [23] and ‘MN55’ (‘Honeycrisp’ x ‘Monark’) [24]. This population was evaluated only in 2016 and was grown at the Horticultural Research Center in a separate location from the first population. Trees from this population were budded onto ‘Budagovsky 9’ rootstock in 2009. An additional 460 genotypes from this family were evaluated for ZLC in a greenhouse at the seedling stage prior to marker assisted culling in 2018.

### Phenotypic data

Trees were rated in 2014, 2015, and 2016 for ZLC on a 1–5 scale similar to that described in Schupp [9], where 0 = no observable ZLC, 1 = up to 25% of leaves affected by clearly expressed ZLC on at least 2 branches, 2 = 25–50% of leaves affected by ZLC, 3 = 50–75% of leaves affected by ZLC, 4 = 75–100% of leaves affected by ZLC (ZLC ratings for all material can be found in S2 Table). Zonal leaf chlorosis ratings were not included if chlorosis due to apple scab (*Venturia inaequalis*) or cedar apple rust (*Gymnosporangium juniperi-virginianae*) could not be distinguished from ZLC. Zonal leaf chlorosis ratings for genotypes were averaged over replicates for use in quantitative trait loci (QTL) analyses.

Seedlings evaluated in 2018 were characterized into 4 classes: extreme dwarf, dwarf, normal, or intermediate at 4 weeks post germination. Extreme dwarf seedlings were less than less than 20 mm tall with extremely small and chlorotic leaves the size of cotyledons and no or undetectable internodes (label “A” in S1 Fig). Dwarf seedlings were less than 25–50 mm tall with short internodes and smaller than normal leaves that exhibited chlorosis and sometimes a rusty color (label “B” in S1 Fig). Normal seedlings were 100–150 mm tall, had 4–8 true leaves that were a healthy green color, and had an active apical meristem with normal leaf initiation and development (label “C” in S1 Fig). Seedlings that were considered intermediate in size were between the classification of normal and dwarf. The intermediate class constituted 3% of the family and were excluded from analysis because it was difficult to determine whether they should be included in the normal or dwarf categories.

Initial results from QTL analyses suggested that a large effect QTL for ZLC was close to a well-documented QTL for fruit skin overcolor attributable to anthocyanin production. In order to determine how close the QTL are to one another and to determine phasing of functional haplotypes, skin color was phenotyped in the families of ‘Honeycrisp’ crossed with ‘Jonafree’, MN1702, ‘Monark’, and ‘Pitmaston Pineapple’ using fruit harvested and stored for concurrent studies in 2015, and from all trees bearing fruit in population 1 in 2016 following the phenotyping protocol used in RosBREED for “Blush/stripe color” [25] (1 = no blush/stripe coverage, 2 = less than 25% blush/stripe coverage, 3 = 25 to 50% blush/stripe coverage, 4 = 50–75% blush/stripe coverage, and 5 = 75–100% blush/stripe coverage.

Year-to-year consistency of phenotype data for ZLC and fruit skin overcolor was evaluated using Kendall’s tau-b coefficient using the Kendall package [26] in the statistical software R version 3.3.0 [27].

### DNA marker data

All individuals evaluated in 2014, 2015, and 2016 were genotyped on the International RosBREED SNP Consortium 8K Illumina Infinium array v1 (Marker data for all genotypes evaluated in this study can be found in S2 Table)[28]. DNA extraction protocols for genotyping on the array were the same as those described in Clark et al. [7]. The marker data generated with the arrays was processed as described in Howard et al., 2017 [22]. The genetic map used in this study is also described in Howard et al., 2017 [22].

Seedlings evaluated in 2018 were genotyped by Ag-Biotech Inc. (Monterey, CA) using Kompetitive Allele Specific PCR (KASP) for SNPs ss475882938, ss475879516, ss475879521, and ss475879580 following the protocol in Simko et al. [29]. DNA extraction used a CTAB protocol from Doyle [30] modified for high throughput. These SNPs were chosen based on segregation data from the individuals genotyped on the 8K SNP array following the QTL analyses and subsequent haplotype analyses. The primer sequences used are included in S3 Table.

### QTL analyses

The QTL analysis methodology used and the rationale for the use of this methodology in this study closely follows that of Howard et al., 2018 [31]. As described in that study, FlexQTL software was used to conduct the QTL analyses. Markov chain Monte Carlo simulation lengths of 2.5*10^5^ were used for each analysis. Every 250^th^ sample was stored for a total of 1000 samples for use in posterior QTL inferences. QTL positions were identified based on QTL intensity estimates via posterior distributions of QTL locations. QTL regions were recorded as consecutive 2 cM bins where each bin had 2*ln Bayes factors [32] that were greater than 5, indicating strong evidence for QTL [33]. QTL peaks were recorded as the median cM value from the Markov chain Monte Carlo simulation samples within the QTL regions.

Separate QTL analyses were performed for each population per year and for ZLC ratings averaged over all three years for the ‘Honeycrisp’ families. Two QTL analyses were performed for each population-year combination using different starting seed numbers for the Markov chain Monte Carlo simulation to ensure the results for the identified QTL and their positions were reproducible (data from only one simulation is reported).

The physical location of SNPs near identified QTL were determined by blasting the 50 bp flanking sequence of each SNP against version 1.1 of the GDDH13 apple reference genome [34] (https://iris.angers.inra.fr/gddh13/) with BLASTN 2.6.0+ [35]. If analyses indicated multiple possible physical locations, the location that corresponded best to the SNP’s position in the genetic map was used.

### Haplotype analysis

QTL that were consistently identified in at least two years and present in the QTL analysis using data averaged over all years with 2*ln Bayes factors greater than 5 were chosen for haplotype analysis. Groups of SNPs at the same cM position containing and bordering the QTL peaks from the QTL analysis using averaged data were used for haplotype analysis. Additional markers flanking the ends of these regions were also included if deemed necessary to differentiate haplotypes. The region included in each haplotype was limited to less than 6 cM to limit recombinant haplotypes in seedling individuals in subsequent analyses. Haplotypes were assigned based on their identities by state (IBS) and traced through known pedigrees to their furthest ancestor. Additional markers not originally included in the genetic map used in this study [22] were included in QTL regions for haplotyping when deemed necessary to help differentiate between identical by state haplotypes. These markers were placed based on physical positions as described in the previous section. The correct placement of markers was validated by ensuring that the inclusion of these markers did not introduce false double recombination events due to incorrect marker placement by evaluating segregation data.

The methodology for comparing haplotype effects among and within families closely follows that of Howard et al., 2018 [31]. The effects of functional haplotypes were estimated based on mean disorder incidence and 95% confidence intervals for each defined haplotype group that were generated from 10,000 bootstrap sample means using R [27]. This method was used because of the extremely non-normal distribution of the phenotypic data and to incorporate the uneven replication in the populations that were evaluated. Each bootstrap mean was comprised of N observations of disorder incidence with replacement, where N was equal to the number of genetically distinct individuals in the dataset from which bootstrap statistics were being generated. The replicates for individuals were weighted, such that the disorder incidence observations for an individual with two replicate trees were individually half as likely to be chosen at random for inclusion into a single bootstrap sample mean compared to an individual represented by a single tree. Conclusions regarding haplotype effects were based on differences between mean ZLC ratings and associated 95% confidence intervals of the defined haplotype groups, without correction for multiple comparisons.

## Results

### Phenotypic data

ZLC ratings tended to be highest in the ‘Honeycrisp’ x ‘Pitmaston Pineapple’ family (S2 Fig). ZLC ratings were also lower in 2015 vs. 2014 and 2016 (S2 Fig). Most ZLC ratings were not as high as ‘Honeycrisp’, which was at the highest rating of 4 each year. Fruit skin overcolor ratings were generally high in most families, with only some seedlings in the ‘Honeycrisp’ x ‘Pitmaston Pineapple’ family having the lowest rating of 1, indicating no red color (S3 Fig). This family had the lowest overall fruit skin overcolor rating and the widest distribution. Relative ZLC rating and fruit skin overcolor rating between seedlings was highly consistent between years (S4 Table).

### QTL analyses

**Fig 1:**
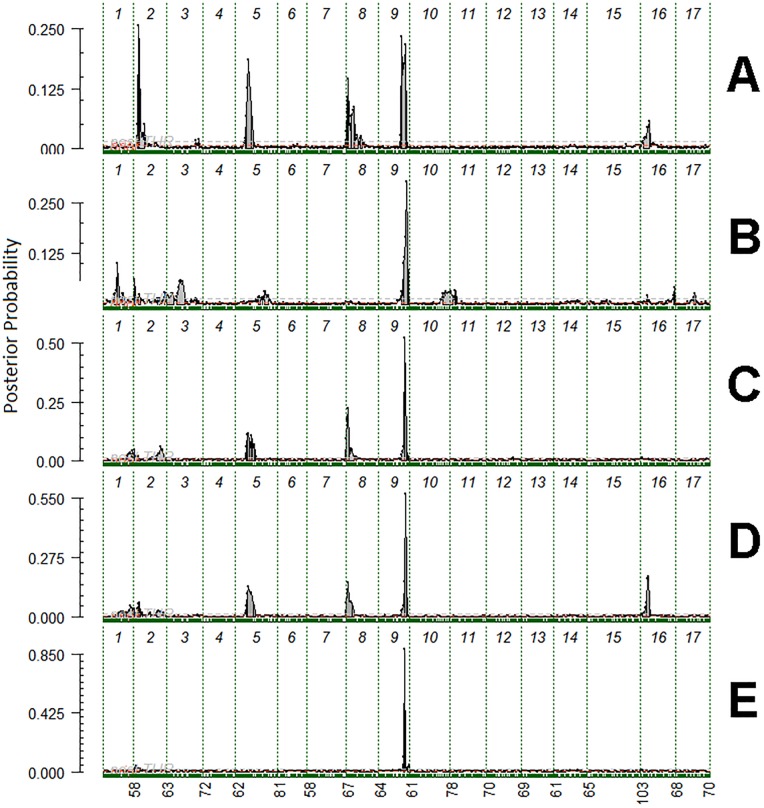
Posterior probability for QTL positions from FlexQTL output for zonal leaf chlorosis for the five ‘Honeycrisp’ families evaluated together in 2014 (A), 2015 (B), 2016 (C), averaged across all years (D), and for the ‘Minneiska’ x ‘MN55’ family evaluated in 2016 (E). Chromosome numbers are indicated at the top of each graph. Green lines at the bottom of each graph represent SNP marker coverage. The numbers below each graph represent cumulative cM position.

One major QTL for ZLC was consistently identified in all QTL analyses near the bottom of linkage group (LG) 9 (Table 1; Fig 1; S5 Table). The peak for this QTL ranges from 49.5 cM to 54.2 cM (Table 1) and from approximately 27.9 Mbp (SNP ss475877680) to 30.5 Mbp (ss475883726). Two smaller QTL were identified on LGs 5 and 8 in both 2014 and 2016, as well as in the QTL analysis using ZLC ratings averaged over all three years (Table 1;Fig 1; S5 Table). The LG5 QTL peaks range from 25.8 cM to 28.0 cM and from approximately 34.7 Mbp to 35.5 Mbp. The LG8 QTL peaks range from 3.8 cM to 9.8 cM and from approximately 2.7 Mbp to 4.9 Mbp.

**Table 1:** QTL regions and peak positions for zonal leaf chlorosis and overcolor rating for QTL with 2*ln Bayes factors greater than 5. The reported QTL regions consist of consecutive 2cM bins with 2*ln Bayes factors greater than 5. QTL peaks were recorded as the median cM value from the Markov chain Monte Carlo simulation samples within the QTL regions.

Trait	Population	Year	LG	QTL region (cM)	QTL peak (cM)
Zonal leaf chlorosis	Five families with ‘Honeycrisp’ as a common parent	2014	2	8–12	9.86
			5	20–32	25.82
			8	2–18	9.79
			9	44–54	49.51
		2015	9	48–58	54.19
		2016	5	20–38	28.01
			8	0–6	3.83
			9	50–56	51.62
		Averaged between 2014–2016	5	22–36	27.58
			8	0–14	5.42
			9	50–56	53.2
	‘MN55’ x ‘Minneiska’	2016	9	48–52	50.3
Overcolor	Four families with ‘Honeycrisp’ as a common parent (‘Honeycrisp’ x MN1764 was not included)	2015	9	56–60	58.74
	Five families with ‘Honeycrisp’ as a common parent	2016	9	30–48	37.58
			9	56–61.06	59.96
	Four families with ‘Honeycrisp’ as a common parent (‘Honeycrisp’ x ‘Pitmaston Pineapple’ excluded)	2016	9	52–61.06	58.55

The location of the LG9 ZLC QTL is close to the previously reported location of a major QTL associated with fruit skin overcolor due to anthocyanin production regulated by the *MdMYB1* gene [36], [37]. ‘Honeycrisp’ is heterozygous at this locus (S6 Table) and its offspring segregate for fruit skin overcolor (S3 Fig). The expected major QTL for skin color at the end of LG9 was confirmed in both 2015 (“A” in S4 Fig) and 2016 (“B” in S4 Fig). An additional smaller QTL for fruit skin overcolor was identified in the middle of LG9 in 2016. This QTL was suspected to be a false QTL due to severe segregation distortion in the ‘Honeycrisp’ x ‘Pitmaston Pineapple’ family coupled with the higher level of phenotypic variance observed in this family (S3 Fig). An additional QTL analysis was performed without this family for the 2016 data and the extra peak was not present (“C” in S4 Fig) and so was not evaluated further. The peak for the consistent QTL for fruit skin overcolor colocalized with *MdMYB1* at 5 to 10 cM from the LG9 QTL for ZLC, depending on the year of evaluation (Table 1).

### Haplotype analysis at the major ZLC QTL on LG9

**Fig 2:**
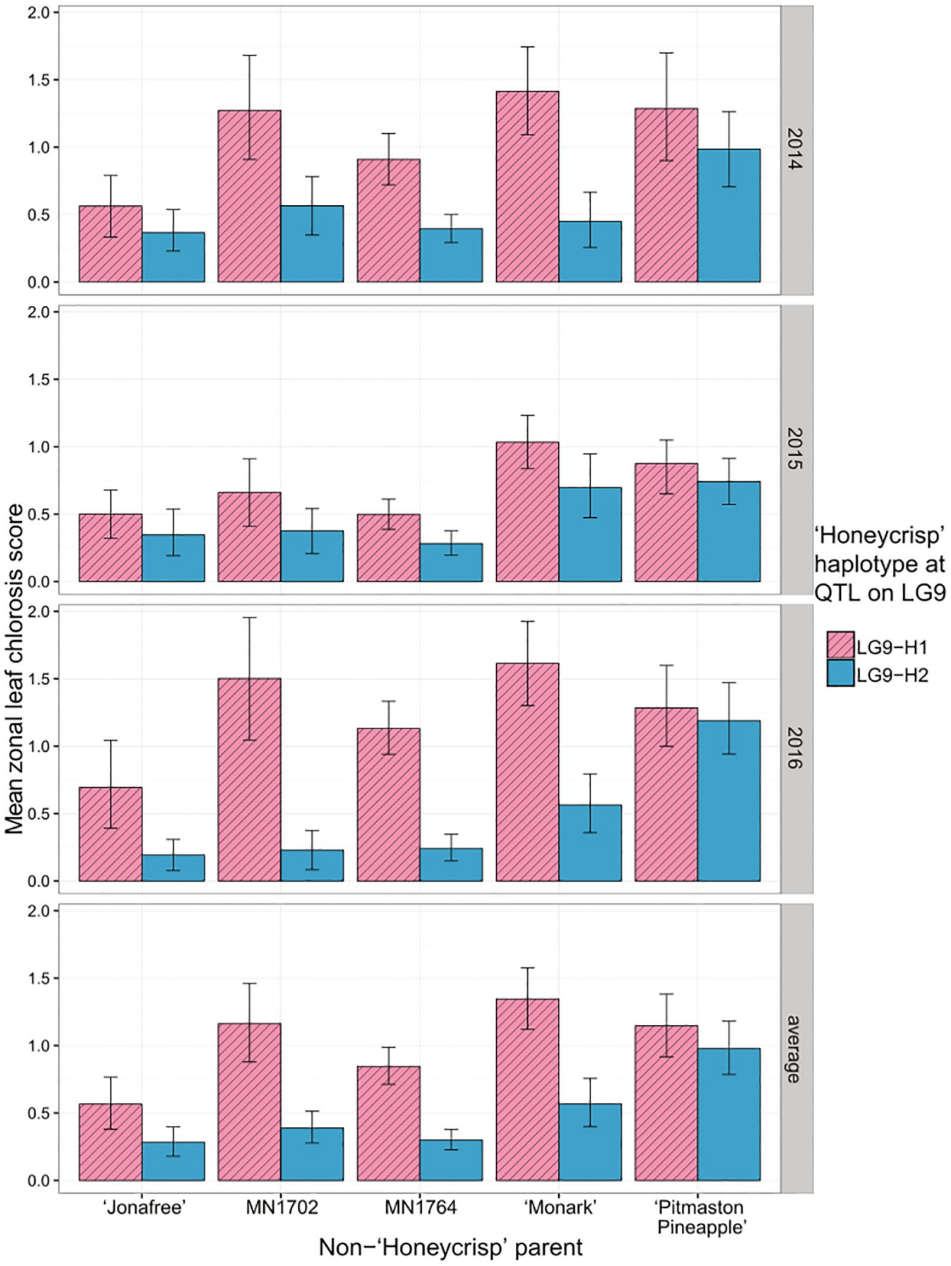
Mean and 95% confidence intervals based on bootstrapping for zonal leaf chlorosis between different partitions of seedling individuals from the ‘Honeycrisp’ families grouped by non-‘Honeycrisp’ parent and the ‘Honeycrisp’ haplotype contribution at the LG9 QTL for zonal leaf chlorosis.

Marker index numbers, names, genetic positions, and physical coordinates used in haplotype analyses are summarized in S3 Table. Ten haplotypes assigned by marker state at the LG9 QTL were represented in seedlings across the ‘Honeycrisp’ families using 18 markers between 50.11 cM and 55.84 cM (S7 Table). One of these markers, ss475879579, was originally not included in the linkage map used in this study, but was deemed useful to differentiate between the LG9-H1 and LG9-H10 haplotypes (S7 Table). The LG9-H1 haplotype (S7 Table) was associated with higher average ZLC ratings in individuals (Fig 2). This trend was consistent in population 1 (Fig 2) and population 2 in each year, though the difference in average ZLC ratings between ‘Honeycrisp’ haplotypes was less pronounced in the ‘Jonafree’ and ‘Pitmaston Pineapple’ families. Bootstrapped mean ZLC ratings with 95% confidence intervals for diplotypes from population 2 were as follows: LG9-H9/LG9-H1–1.38 (1.07–1.69), LG9-H1/LG9-H5–0.90 (0.67–1.14), LG9-H9/LG9-H5–0.10 (0.02–0.20).

**Fig 3:**
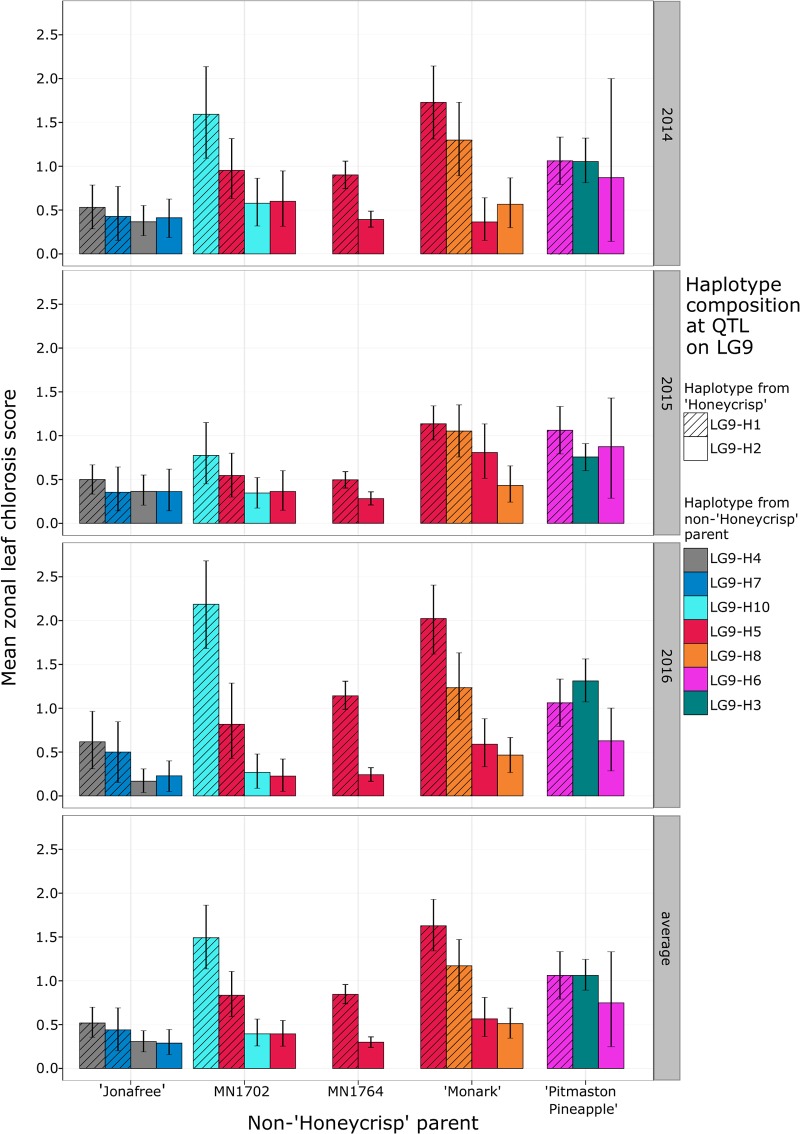
Mean and 95% confidence intervals based on bootstrapping for zonal leaf chlorosis between different partitions of seedling individuals from the ‘Honeycrisp’ families grouped by non-‘Honeycrisp’ parent and diplotype at the LG9 QTL for zonal leaf chlorosis.

Diplotypes LG9-H1 + LG9-H10 in the ‘Honeycrisp’ x MN1702 family and LG9-H1 + LG9-H5 in the ‘Honeycrisp’ x ‘Monark’ family were associated with higher ZLC ratings than the alternate diplotypes within those families in some years (Fig 3). This difference was most apparent in 2016.

The LG9-H1 haplotype is a recombinant haplotype that ‘Honeycrisp’ inherited from its grandparent ‘Duchess of Oldenburg’ (S7 Table) through parent MN1627 [22]. The recombination of the ‘Duchess of Oldenburg’ haplotypes (LG9-H11 and LG9-H5, S7 Table) occurred between two groups of markers that each have the same cM. One haploblock is at 52.23 cM and contains SNPs ss475879578, ss475882948, ss475883726, and ss475879580. The other haploblock is at 55.8 cM and contains SNPs ss475882939, ss475879523, and ss475879524. The area between these haploblocks contains the QTL peak identified in 2015, 2016, and the QTL analysis using ZLC ratings averaged across all three years for the five ‘Honeycrisp’ families, though not the QTL peak for the ‘Minneiska’ x ‘MN55’ family (Table 1). To determine which ‘Duchess of Oldenburg’ haplotype was associated with the high ZLC phenotype observed in ‘Honeycrisp’, other individuals that inherited either haplotype from ‘Duchess of Oldenburg’ were identified in available germplasm.

The LG9-H5 haplotype that MN1702 inherited from ‘Frostbite’ was inherited from ‘Duchess of Oldenburg’, as ‘Duchess of Oldenburg’ is one grandparent of ‘Frostbite’ [22] and the entirety of LG9 in ‘Frostbite’ is composed of ‘Duchess of Oldenburg’ haplotypes, with evidence for a recombination having occurred at approximately 30 cM (in the map used in this study). This would make the last three markers of the LG9-H5 haplotype identical by descent with of the LG9-H1 haplotype in ‘Honeycrisp’ (S7 Table). Progeny of MN1702 that inherited the LG9-H5 haplotype were found to have a significantly higher ZLC rating compared to progeny that inherited the LG9-H10 haplotype in 2016, but only when coupled with the LG9-H1 haplotype from ‘Honeycrisp’ (Fig 2).

MN1764 is homozygous for the LG9-H5 haplotype. Although pedigree records are not available for MN1764, this selection likely inherited both haplotypes from ‘Duchess of Oldenburg’ considering the prevalence of ‘Duchess of Oldenburg’ in the pedigrees of UMN breeding selections and because MN1764 is homozygous for a ‘Duchess of Oldenburg’ haplotype for 28.0 cM of LG9 (including the LG9 QTL region for ZLC), as well as for the first 24.6 cM and last 22.0 cM of LG12 and because MN1764 contains multiple long haplotypes that are identical by state with haplotypes in ‘Duchess of Oldenburg’. MN1764 itself does not exhibit ZLC and the ‘Honeycrisp’ x MN1764 family is not associated with higher average ZLC rating compared to other families evaluated in this study.

‘Monark’ also likely inherited its LG9-H5 haplotype from ‘Duchess of Oldenburg’, as one homolog of LG9 in ‘Monark’ consists of haplotypes from ‘Duchess of Oldenburg’ from 16.8 cM to the end of the LG, with a recombination between 41.5 cM and 42 cM. Similarly long haplotypes that are identical between both cultivars exist on LGs 2, 5, 6, 10, and 17, indicating ‘Duchess of Oldenburg’ is a likely ancestor of the chronologically much more recent ‘Monark’. Progeny of ‘Monark’ that inherited the LG9-H5 haplotype were found to have a significantly higher ZLC rating compared to progeny that inherited the LG9-H8 haplotype in 2016, but only when coupled with the LG9-H1 haplotype from ‘Honeycrisp’ (Fig 3).

Several cultivars released from the UMN apple breeding program contain one copy of the LG9-H5 haplotype from ‘Duchess of Oldenburg’, including ‘Haralson’, ‘Regent’, and ‘Red Baron’, as well as multiple UMN breeding selections, but no cultivars or selections have been identified with the LG9-H11 from ‘Duchess of Oldenburg’, which would correspond with the first 15 markers of the LG9-H1 haplotype associated with higher ZLC incidence.

### Segregation distortion at ZLC QTL on LG9

Segregation distortion at the LG9 QTL was observed in two families. The ‘Honeycrisp’ x ‘Pitmaston Pineapple’ family was deficient of individuals with the LG9-H1 haplotype. This genomic region exhibited the most severe segregation distortion of anywhere across the genome for ‘Honeycrisp’ haplotypes in this family. Additionally, although the diplotype of ‘Pitmaston Pineapple’ is LG9-H3 + LG9-H6 (S7 Table), the ‘Pitmaston Pineapple’ family contained no individuals with the LG9-H1 + LG9-H3 diplotype and only three individuals with the LG9-H2 + LG9-H6 diplotype. The first 15 markers of the LG9-H3 haplotype are identical to those of the LG9-H1 haplotype. This area contains the QTL peaks from each QTL analysis. Furthermore, the area shared between ‘Pitmaston Pineappe’ and ‘Duchess of Oldenburg’ that contains the 15 marker shared portions of the LG9-H3 and LG9-H1 haplotypes in these cultivars, respectively, extends from 41.5 cM to 53.6 cM, indicating that these haplotypes could have come from the same, but unknown, pedigree ancestor.

The ‘Minneiska’ x ‘MN55’ family evaluated in the field in 2016 contained no individuals with two copies of the LG9-H1 haplotype, though an approximately equal proportion of individuals with the other three diplotype combinations was observed. Seed germination and seedling propagation records indicated that a higher percentage of individuals from this family and other families where both parents were heterozygous for LG9-H1 were stunted and either died or were culled due to lack of vigor several weeks after germination when compared to other families in the breeding program.

Of the 460 seedlings from the ‘Minneiska’ x ‘MN55’ cross evaluated in the greenhouse in 2018, 380 lived to the 4-week post germination period. Data for 44 seedlings was discarded for having failed SNP call test results and 10 were discarded for being in the intermediate size category. Of the remaining 326 seedlings, 60 of them were categorized as dwarf or extreme dwarf. Three of the four SNPs used for genotyping the seedlings had consistent genotype ratings. SNP ss475879580 had far more heterozygous SNP calls than expected. These SNP calls were inconsistent with the other three SNPs used for genotyping the seedlings and thus were excluded from analysis. The genotype ratings for seedlings characterized as dwarf and extreme dwarf were similar, so their genotype ratings were combined for comparison to the genotype ratings for seedlings characterized as normal. Few of the seedlings characterized as normal were homozygous LG9-H1, whereas all individuals characterized as dwarf or extreme dwarf were homozygous LG9-H1 (Table 2).

**Table 2:** Number of seedlings from a ‘Minneiska’ x ‘MN55’ cross observed in each genotype class for 3 SNPs in the LG9 ZLC QTL and in normal and dwarf phenotype classes in 2018.

Seedling characterization	Copies of the LG9-H1 haplotype associated with ZLC	Number of seedlings within each genotype class for 3 SNPs at the LG9 QTL for ZLC		
		ss475882938	ss475879516	ss475879521
Normal	0	8	6	0
	1	187	185	195
	2	71	75	70
Dwarf or Extreme dwarf	0	49	49	53
	1	8	8	7
	2	0	0	0

### Haplotype analysis at QTL on LG9 for skin overcolor

Five different haplotypes were observed at the location of the LG9 QTL for fruit skin overcolor (S6 Table). ‘Honeycrisp’ and ‘Pitmaston Pineapple’ were the only parents that segregated for haplotypes at this locus. Severe segregation distortion prevented the identification of clear effects of haplotypes from ‘Pitmaston Pineapple’. The haplotype ‘Honeycrisp’ inherited from grandparent ‘Duchess of Oldenburg’ had clear and consistently higher average skin overcolor rating in all families compared to the other haplotype inherited from grandparent ‘Frostbite’ (S5 Fig). Both this haplotype, and the LG9-H1 haplotype associated with increased ZLC rating, are from ‘Duchess of Oldenburg’ through parent MN1627 and are in coupling phase (Howard et al., 2017 [22]). Thus, the red haplotype at the skin overcolor QTL in ‘Honeycrisp’ is linked in coupling phase with the haplotype associated with higher average ZLC rating at the LG9 ZLC QTL.

### Haplotype analysis at QTL for ZLC on LGs 5 and 8

The LG5-H4 haplotype in ‘Pitmaston Pineapple’ (S8 Table) at the LG5 QTL was associated with higher ZLC ratings in 2015 and 2016 (S6 Fig). The LG8-H1 haplotype in ‘Honeycrisp’ at the LG8 QTL (S9 Table) inherited from grandparent ‘Northern Spy’ through parent ‘Keepsake’ was associated with higher ZLC ratings in the ‘Monark’ x ‘Honeycrisp’ family when paired with the LG9-H1 haplotype in 2015 and 2016 (S7 Fig). No other clear trends were observed between average ZLC ratings and other haplotypes at the LG5, LG8, or LG9 QTL.

## Discussion

### Major QTL for ZLC on LG9

A major QTL for ZLC was identified on LG9 in all years of evaluation in this study (Table 1; Fig 1; S5 Table), making this the first study to identify a genetic basis for this disorder. The LG9-H1 haplotype (S7 Table) from ‘Honeycrisp’ was associated with higher average ZLC ratings in all families and all years evaluated (Fig 2). The LG9 QTL provides a region to target for fine mapping and candidate genes that might be involved with the hypothesized physiological basis for ZLC. Combining knowledge of this QTL location with other data (e.g. differential expression studies) could direct future studies aimed at understanding the carbohydrate transport system, introducing specific hypotheses that were not conceivable in previous studies of ZLC in ‘Honeycrisp’ [10], [14], [15].

### Genetics of ZLC incidence underlying identified QTL

The genetics and expression of ZLC in ‘Honeycrisp’ and its progeny were not explained solely by the LG9-H1 haplotype at the LG9 QTL for ZLC incidence. The majority of the ‘Honeycrisp’ progeny with the LG9-H1 haplotype did not express ZLC at the same severity as ‘Honeycrisp’, which was consistently rated a 4 in each year for the two trees grown alongside its progeny evaluated in this study. It is possible that the specific diplotype of ‘Honeycrisp’ at the LG9 QTL for ZLC is responsible for its high level of ZLC. Some evidence for other alleles at the LG9 QTL for ZLC influencing severity of the disorder can be found in the ‘Honeycrisp’ x MN1702, ‘Honeycrisp’ x ‘Monark’, and the ‘Honeycrisp’ x ‘Pitmaston Pineapple’ families. In the ‘Honeycrisp’ x MN1702 and ‘Honeycrisp’ x ‘Monark’ families, the LG9-H1 + LG9-H5 diplotype was found to be associated with higher average ZLC ratings, particularly in 2016 where ZLC ratings for this diplotype were significantly higher (Fig 3). Severe segregation distortion was observed at the LG9 QTL for ZLC in the ‘Honeycrisp’ x ‘Pitmaston Pineapple’ family, with no individuals having the LG9-H1 + LG9-H3 diplotype and very few individuals with the diplotype LG9-H2 + LG9-H6 present for evaluation in the field. It is possible that the LG9-H3 haplotype in ‘Pitmaston Pineapple’ is identical by descent with the first portion of the LG9-H1 haplotype, as evidenced by both haplotypes sharing the first 15 markers of their haplotypes as well as 10cM upstream from that general position. The first portion of the area used for haplotyping containing those 15 shared markers contains the QTL peaks for ZLC (Table 1). If those haplotypes are truly identical by descent, the lack of individuals with the LG9-H1 + LG9-H3 diplotype present in the field would be consistent with the hypothesis posed with the ‘Minneiska’ x ‘MN55’ family that two copies of the deleterious genetics underlying the LG9-H1 haplotype is seedling lethal. The overall lack of individuals with the LG9-H1 haplotype and the LG9-H2 + LG9-H6 diplotype in this family also suggest that the other haplotype from ‘Pitmaston Pineapple’ may also play a role in ZLC formation. Additionally, the specific diplotypes possessed by ‘Honeycrisp’ at the smaller effect QTLs identified on LGs 5 (S4 Fig) and 8 (S5 Fig), which were only relevant in particular families, may also contribute to more severe ZLC expression. Genotype by year interactions also played a role in ZLC severity in ‘Honeycrisp’ progeny as the QTLs on LGs 5 and 8 were not identified in 2015. This year was also marked by a lower average population-wide ZLC rating compared to 2014 and 2016. The presence of year effects on ZLC severity have also been observed in studies involving ‘Honeycrisp’ [10], [12], [13]. Researchers in future studies of ZLC should prudently evaluate trees in multiple years or locations or under varying management regimes based on this evidence of interaction of genotype with as yet unknown environmental factors.

### Breeding implications of the major QTL on LG9

The observation of severe segregation distortion disfavoring the LG9-H1 haplotype from ‘Honeycrisp’ associated with higher ZLC ratings in offspring indicates a detrimental effect of ZLC and should be considered in breeding decisions, particularly when both parents hold one copy of the haplotype. We deduce this because of the observed segregation distortion in the ‘Honeycrisp’ x ‘Pitmaston Pineapple’ family, because there were no individuals from the ‘Minneiska’ x ‘MN55’ family under field observation that possessed two copies of the LG9-H1 haplotype, and because most seedlings evaluated in 2018 with two copies of the LG9-H1 haplotype were severely dwarfed (Table 2), will likely not grow to be healthy trees, and will therefore be culled. The difference between healthy and dwarf seedlings (S1 Fig) is similar to the difference that has been documented between healthy seedlings and seedlings with dwarfing caused by sub-lethal genes associated with V_f_ apple scab resistance [38]. These results suggest that two copies of the LG9-H1 haplotype is sub-lethal at the seedling level.

The linkage observed between the LG9 QTL for ZLC and the major QTL for skin overcolor on LG9 (Table 1) may also influence breeding decisions. The location of the LG9 QTL for skin overcolor is consistent with other studies [36], [37]. The MYB transcription factor gene, *MdMYB1*, which has been demonstrated to regulate anthocyanin biosynthesis in apple skin color [36], [39] has been suggested as the causative gene underlying this QTL [36], [40], [41]. *MdMYB1* (MDP0000259614) is located on linkage group 9 between 35,545,015 and 35,549,069 bp [37] of version 1.1 of the GDDH13 apple reference genome [34]. The physically closest SNP, ss475879555, is located at 29,470,268 bp (5,610 bp difference) and is at 60.13 cM in the linkage map used in this study [22]. The distances between this SNP and the QTL peaks we identified for overcolor are 1.39 cM and 0.17 cM for 2015 and 2016 (Table 1), respectively. This places *MdMYB1* as close as 5.92 cM or as far as 10.6 cM away from the QTL peaks identified for ZLC (Table 1). The ‘Honeycrisp’ haplotype associated with the red skin overcolor is in coupling phase with the LG9-H1 haplotype associated with increased ZLC. Because red skin overcolor is often under positive selection pressure in apple breeding programs, this linkage could pose a problem particularly in crosses between ‘Honeycrisp’ and a parent that has no red skin overcolor. Most seedlings selected for red skin overcolor will also inherit the LG9-H1 haplotype for ZLC from ‘Honeycrisp’.

The LG9 QTL for ZLC would be a challenging target for marker assisted seedling selection. In addition to complicating selection for red skin color in ‘Honeycrisp’ progeny as described above, the LG9-H1 haplotype in ‘Honeycrisp’ would also be logistically difficult to select against because it is identical by state with haplotypes from other cultivars not known to be associated with ZLC. For example, LG9-H1 haplotype is identical by state with haplotypes in ‘Northern Spy’, ‘Fameuse’, ‘Northwest Greening’ and possibly other cultivars. Also, the uncertainty as to which portion of the recombinant ‘Duchess of Oldenburg’ haplotype that ‘Honeycrisp’ inherited is associated with the disorder further complicates matters, though the QTL was consistently located either within, or closer to, the first portion of the recombinant haplotype (Table 2; S7 Table).

Predicting which cross combinations will result in high ZLC may be difficult. In our study, we identified haplotypes in ‘Duchess of Oldenburg’ that are associated with high levels of ZLC (Figs 1 and 2), but ‘Duchess of Oldenburg’ itself has not been noted in the literature as being prone to the disorder. It is possible that ‘Duchess of Oldenburg’ carries the ZLC genetic disorder in the LG9-H11 haplotype, but does not express the disorder because of the lack of additional genetic factors, or reasons not identified in this study. Indeed, ‘Honeycrisp’ contains a unique diplotype at this locus, and its other haplotype, LG9-H2, possibly combines detrimentally with LG9-H1, resulting in high ZLC. This might explain why parents ‘MN55’ and ‘Minneiska’ have not been noted to exhibit ZLC in production, despite carrying the LG9-H1 haplotype. However, minor ZLC was noted on both ‘MN55’ and ‘Minneiska’ in 2016. Additionally, the hypothesis that ZLC is expressed more strongly in particular diplotypes is supported by the observation that individuals in the ‘Honeycrisp’ x MN1702 and ‘Honeycrisp’ x ‘Monark’ families with the LG9-H1 + LG9-H5 diplotype were associated with higher ZLC (Fig 3) relative to alternative diplotypes in those families. However, this would not explain why ‘Duchess of Oldenburg’ does not exhibit ZLC symptoms, as both the LG9-H1 and the LG9-H5 haplotypes were inherited from ‘Duchess of Oldenburg’. Additional research would be needed to clarify this circumstance. We should note too that minor ZLC was observed on multiple trees of ‘MN55’ and ‘Minneiska’ at the UMN Horticultural Research Center in Chanhassen, Minnesota, USA, in 2016.

Other cultivars may carry the same deleterious allele that is in the LG9-H1 haplotype in ‘Honeycrisp’. We hypothesize that the LG9-H3 haplotype in ‘Pitmaston Pineapple’ and the LG9-H1 haplotype in ‘Honeycrisp’ carry the same genetic factor associated with high ZLC and that both inherited the deleterious allele from the same unidentified pedigree ancestor. The identification of any additional cultivars carrying this allele would be useful when planning crossing schemes.

### Conclusion

The consistent location of the LG9 QTL for ZLC and the consistent effect of the LG9-H1 haplotype from ‘Honeycrisp’ in its offspring indicate this genomic region is a good target for further inquiries into the genetics and physiology of ZLC. Several breeding implications were identified for the QTL, including segregation distortion, seedling lethality of two copies of the LG9-H1 haplotype, linkage between the LG9 QTL for ZLC and the LG9 QTL for skin overcolor, and coupling between the high ZLC and red skin overcolor haplotypes in ‘Honeycrisp’. These implications should be considered in apple breeding programs that are increasingly reliant on ‘Honeycrisp’ and derivative germplasm for highly crisp fruit texture.

## Supporting information

S1 Table Biparental families and sample sizes evaluated in total and per year for QTL analyses for zonal leaf chlorosis. Numbers in parentheses represent the total number of genotypes and their replicates evaluated.
(XLSX)

S2 Table SNP and phenotype data used in this study. (XLSX)

S3 Table SNP names, NCBI dbSNP accession numbers, physical positions on the ‘Golden Delicious’ double haploid genome, and cM positions on the genetic map used in this study for SNPs used for haplotype analysis at QTL identified for zonal leaf chlorosis on LGs 5, 8, and 9 and Kompetitive primer sequence information for SNPs used for analysis of greenhouse seedlings in 2018. (XLSX)

S4 Table Squared Kendall’s tau-b correlation coefficient and p-values for comparisons between 2014, 2015, and 2016 for ZLC incidence and between 2015 and 2016 for fruit skin overcolor rating. (XLSX)

S5 Table Bayes factors (2*ln) for a 1 QTL vs. 0 QTL model per linkage group for ZLC rating, grouped by population and year(s) evaluated. The 2*ln Bayes factors are interpreted as having hardly any (0–2), positive (2–5), strong (5–10), and decisive (>10) evidence for a 1 QTL model vs. a 0 QTL model per linkage group. The darker colors on this table represent the larger Bayes factors.
(XLSX)

S6 Table Haplotype identities assigned by state with founding sources (if known) for each haplotype at the LG9 fruit skin overcolor QTL for all parents of families in this study. Physical and genetic coordinates for each SNP can be found in S3 Table.
(XLSX)

S7 Table Haplotype identities assigned by state with founding sources (if known) for each haplotype at the LG9 zonal leaf chlorosis QTL for all parents of families in this study. Physical and genetic coordinates for each SNP can be found in S3 Table.
(XLSX)

S8 Table Haplotype identities assigned by state with founding sources (if known) for each haplotype at the LG5 zonal leaf chlorosis QTL for all parents of families in this study. Physical and genetic coordinates for each SNP can be found in S3 Table.
(XLSX)

S9 Table Haplotype identities assigned by state with founding sources (if known) for each haplotype at the LG8 zonal leaf chlorosis QTL for all parents of families in this study. Physical and genetic coordinates for each SNP can be found in S3 Table.
(XLSX)

S1 Fig Seedlings from a cross between ‘Minneiska’ and ‘MN55’ characterized as extreme dwarf (A), dwarf (B), and normal (C). (JPG)

S2 Fig Boxplots of ZLC ratings by family and year.laasdfjklsdfre also lower in 2015 vs. 2014 and 2016.tical software R version 3.3.0 ()ll’der incidence was the severe segregation. (PDF)

S3 Fig Boxplots of fruit skin overcolor rating distributions by family and year. (PDF)

S4 Fig Posterior probability for QTL positions from FlexQTL output for overcolor rating in the five ‘Honeycrisp’ families evaluated in 2015 (A) and 2016 (B) and again in 2016 without the ‘Honeycrisp’ x ‘Pitmaston’ family (C). Chromosome numbers are indicated at the top of each graph. Green lines at the bottom of each graph represent SNP marker coverage. Numbers below the series of graphs indicate the cM position at the end of each linkage group.
(PNG)

S5 Fig Mean and 95% confidence intervals for fruit skin overcolor between partitions of seedling individuals from the ‘Honeycrisp’ families grouped by non-‘Honeycrisp’ parent and ‘Honeycrisp’ haplotype contribution at the LG9 QTL for fruit skin overcolor in 2015 and 2016 (bootstrapped data). (PDF)

S6 Fig Mean and 95% confidence intervals for zonal leaf chlorosis between partitions of seedling individuals from the ‘Honeycrisp’ families grouped by non-‘Honeycrisp’ parent, ‘Honeycrisp’ haplotype contribution at the LG9 QTL, and the non-‘Honeycrisp’ haplotype contribution at the LG5 QTL for zonal leaf chlorosis in all years evaluated and averaged across all years evaluated (bootstrapped data). (PDF)

S7 Fig Mean and 95% confidence intervals for zonal leaf chlorosis between partitions of seedling individuals from the ‘Honeycrisp’ families grouped by non-‘Honeycrisp’ parent and ‘Honeycrisp’ haplotype contributions at the LG8 and LG9 QTL for zonal leaf chlorosis in all years evaluated and averaged across all years evaluated (bootstrapped data). (PDF)
