# Correction: Genetics of zonal leaf chlorosis and genetic linkage to a major gene regulating skin anthocyanin production (*MdMYB1*) in the apple (*Malus* × *domestica*) cultivar Honeycrisp

**DOI:** 10.1371/journal.pone.0213799

**Published:** 2019-03-07

**Authors:** Nicholas P. Howard, John Tillman, Stijn Vanderzande, James J. Luby

There are errors in the final sentence of the fourth paragraph beneath the “Haplotype analysis at the major ZLC QTL on LG9” subheading in the Results section. The correct sentence is: "Progeny of MN1702 that inherited the LG9-H10 haplotype were found to have a significantly higher ZLC rating compared to progeny that inherited the LG9-H5 haplotype in 2016, but only when coupled with the LG9-H1 haplotype from ‘Honeycrisp’ (Fig 3)."

There are errors in Table 2. The values in the “Copies of the LG9-H5 haplotype with ZLC” column appear incorrectly. Please see the complete, correct Table 2 here.

**Table 2:** Number of seedlings from a ‘Minneiska’ x ‘MN55’ cross observed in each genotype class for 3 SNPs in the LG9 ZLC QTL and in normal and dwarf phenotype classes in 2018.

Seedling characterization	Copies of the LG9-H1 haplotype associated with ZLC	Number of seedlings within each genotype class for 3 SNPs at the LG9 QTL for ZLC		
		ss475882938	ss475879516	ss475879521
Normal	2	8	6	0
	1	187	185	195
	0	71	75	70
Dwarf or Extreme dwarf	2	49	49	53
	1	8	8	7
	0	0	0	0

The fifth sentence in the second paragraph beneath the “Breeding implications of the major QLT on LG9” subheading in the Discussion section is incorrect. The correct sentence is: "The physically closest SNP, ss475879555, is located at 35,556,722 bp (7,653 bp difference) and is at 60.13 cM in the linkage map used in this study [22]."
