# Supplementary figures and images for: Genetics of zonal leaf chlorosis and genetic linkage to a major gene regulating skin anthocyanin production (MdMYB1) in the apple (Malus × domestica) cultivar Honeycrisp
Source: PLoS One. 2019 Jan 28;14(1):e0210611. doi: 10.1371/journal.pone.0210611 (PMC6349313; doi:10.1371/journal.pone.0210611)

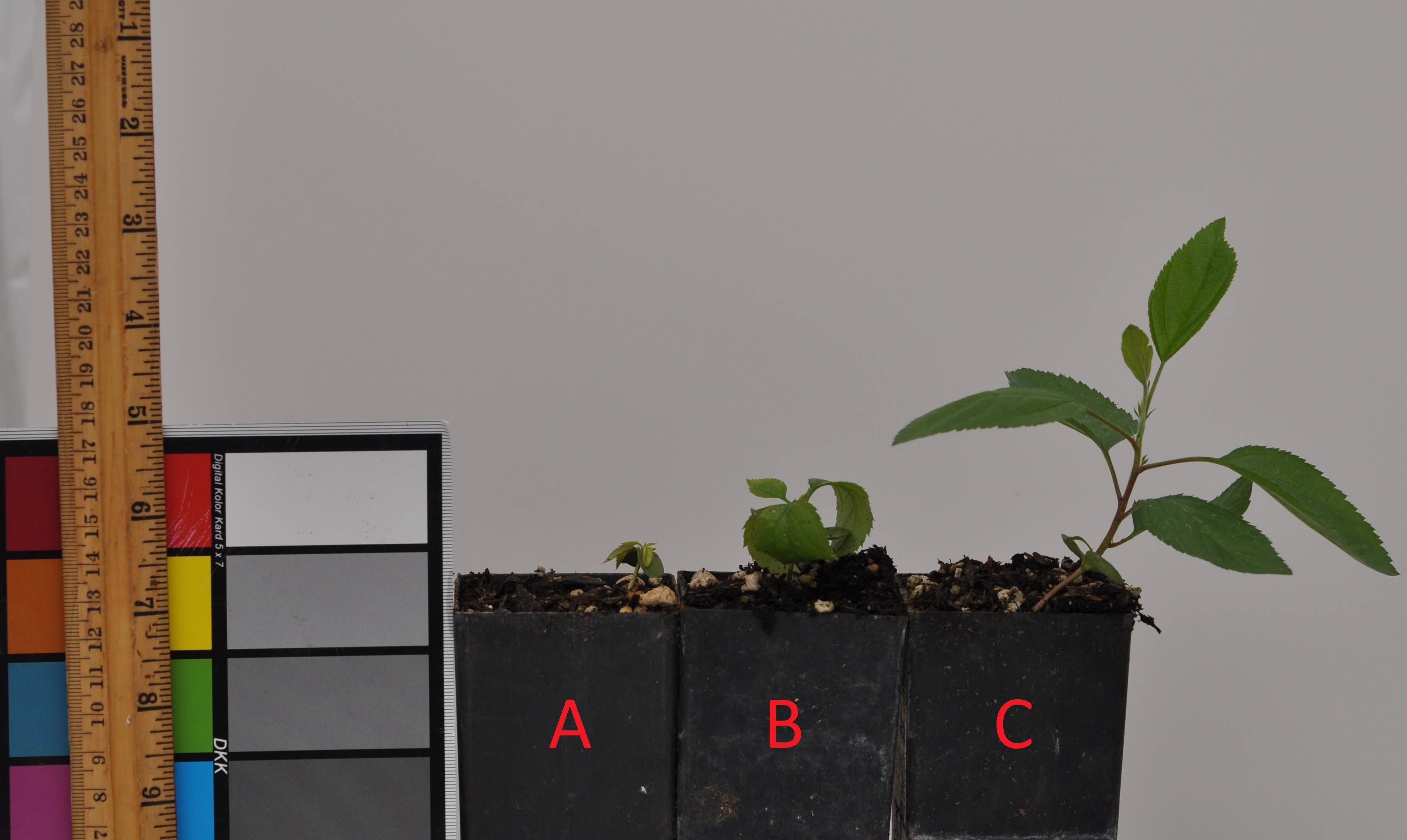

Supplement: S1 Fig — (JPG) [file pone.0210611.s010.jpg]

ZLC rating

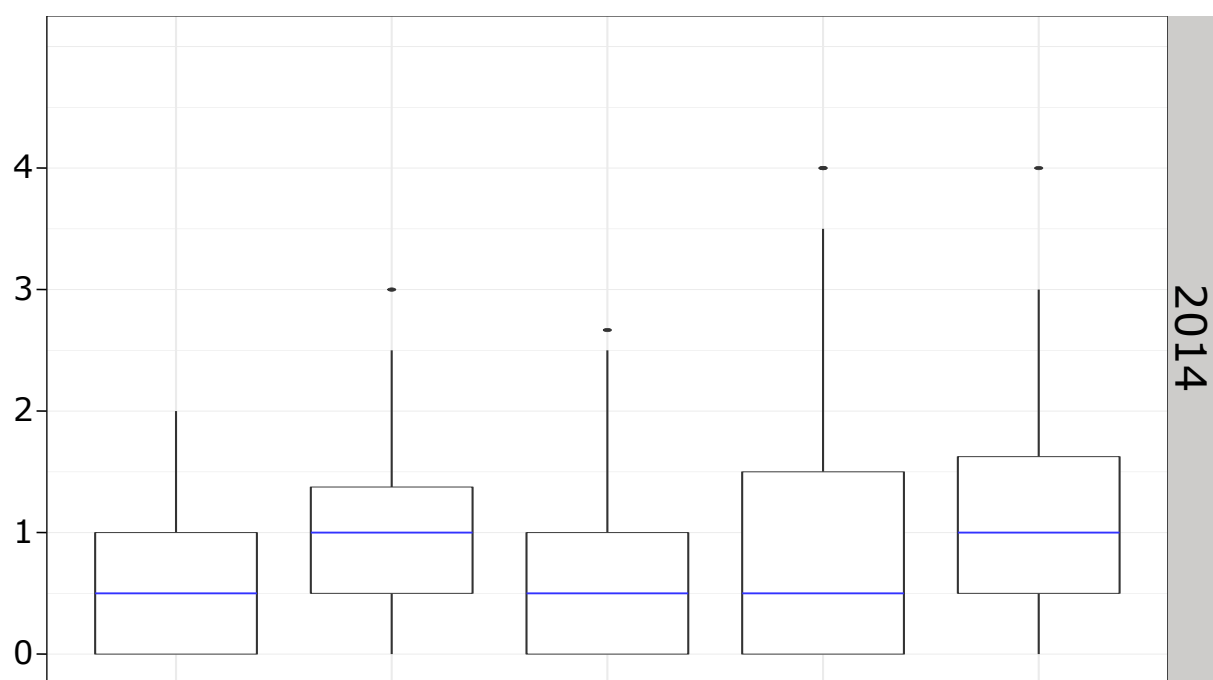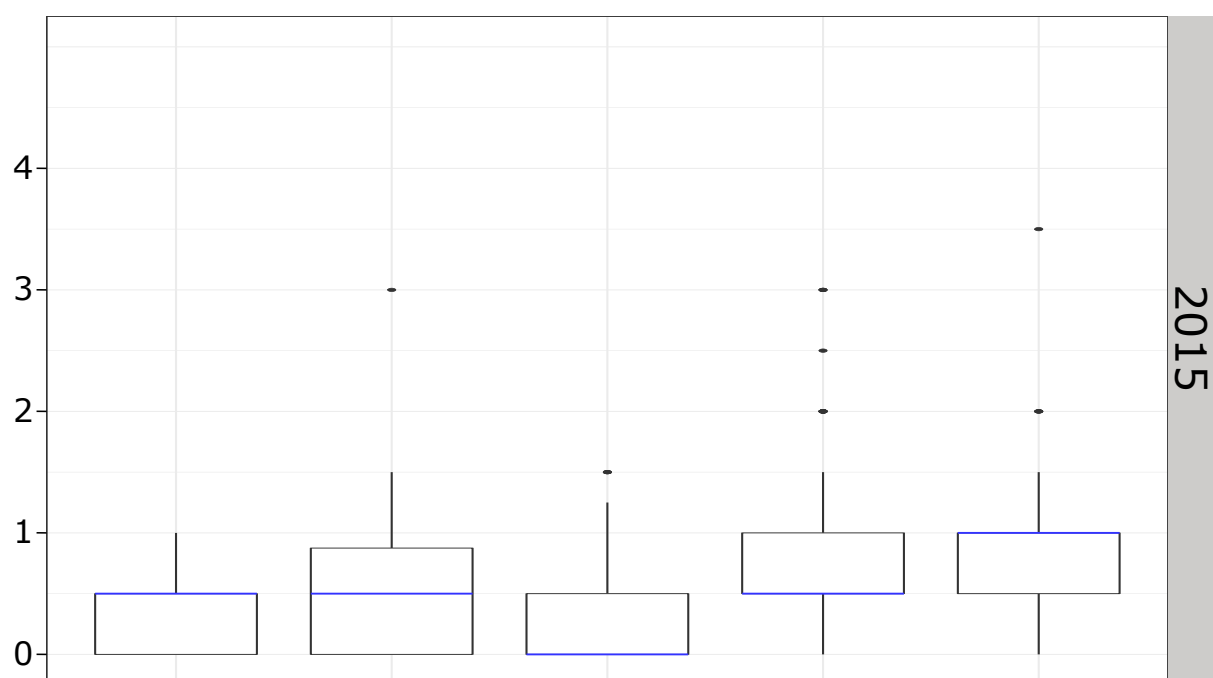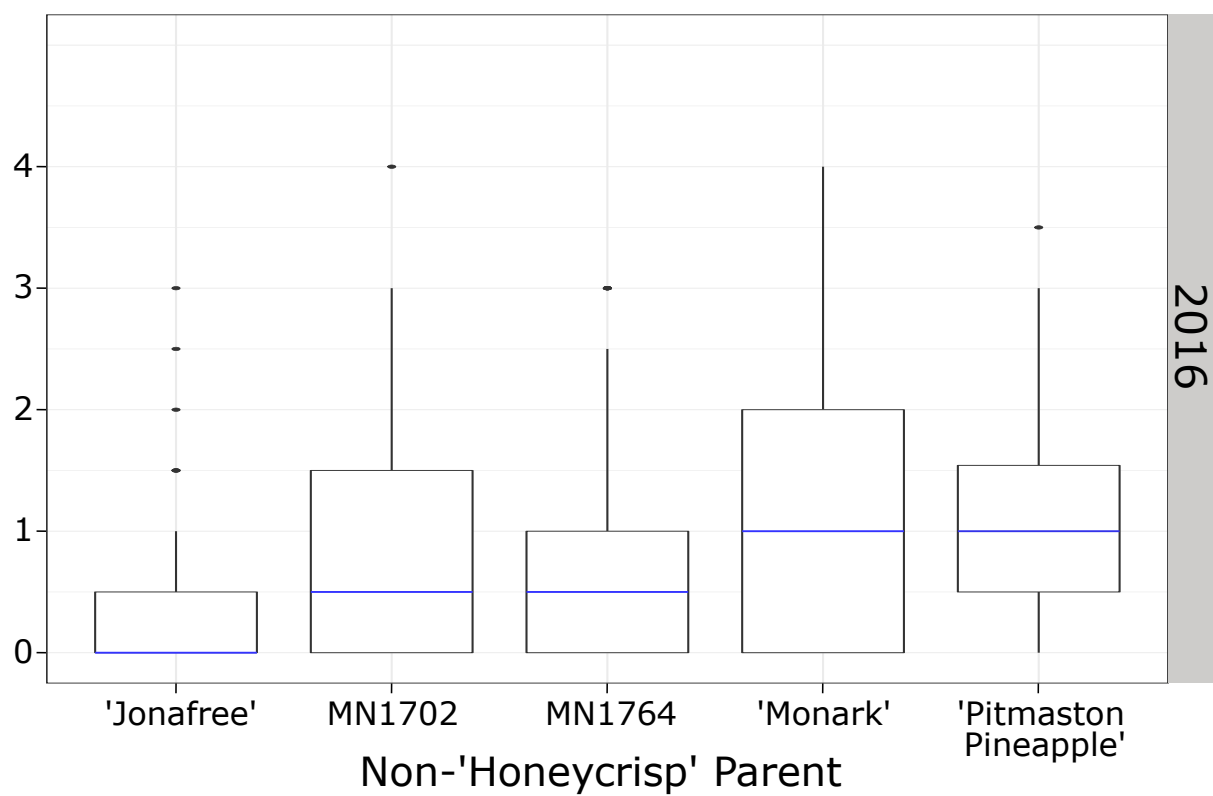

Supplement: S2 Fig — (PDF) [file pone.0210611.s011.pdf]

Skin overcolor rating

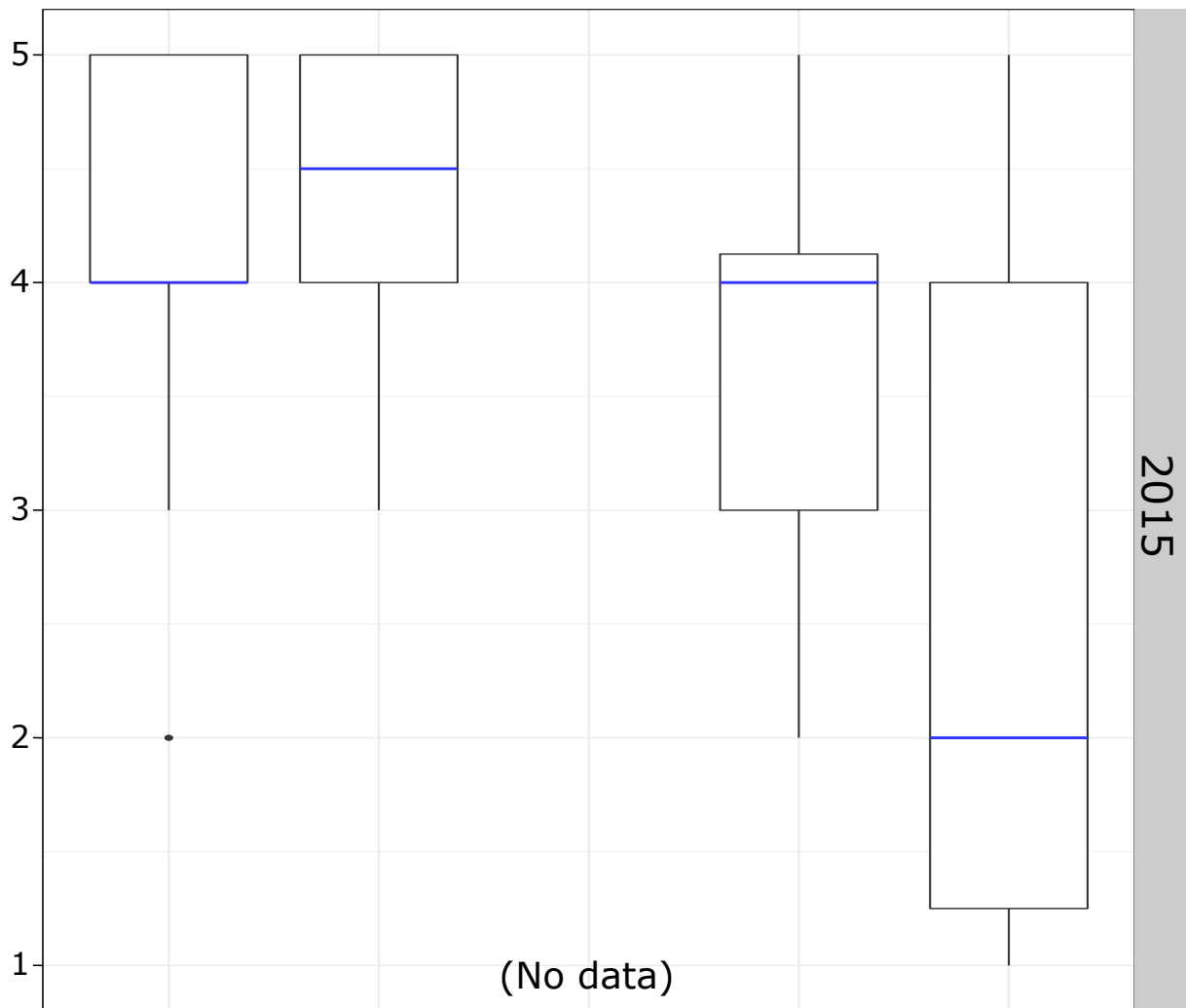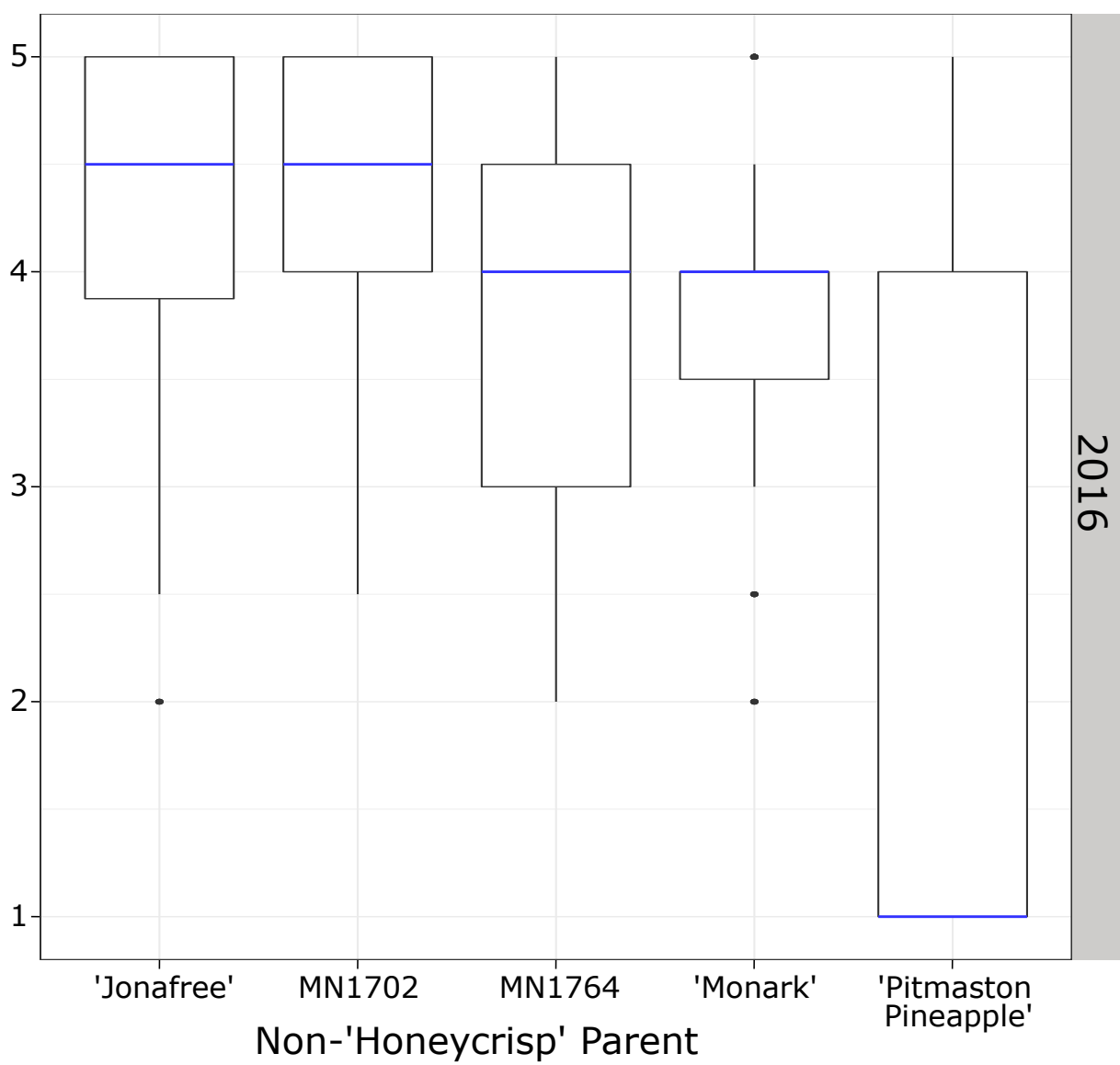

Supplement: S3 Fig — (PDF) [file pone.0210611.s012.pdf]

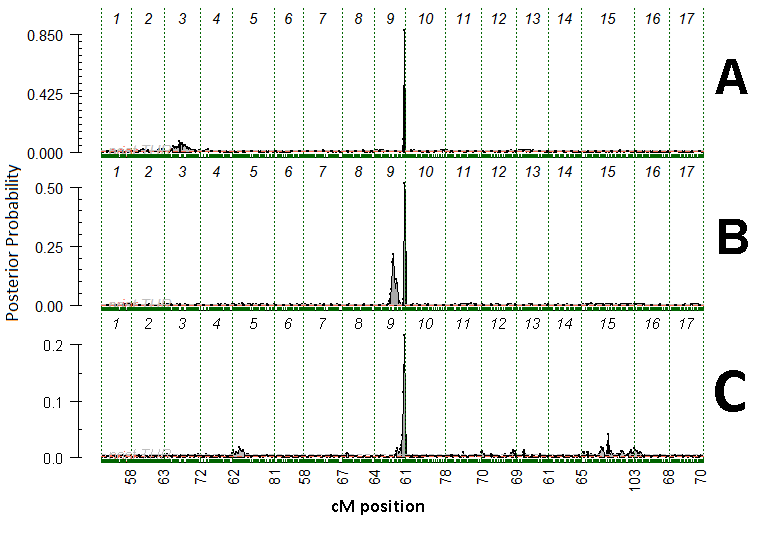

Supplement: S4 Fig — Chromosome numbers are indicated at the top of each graph. Green lines at the bottom of each graph represent SNP marker coverage. Numbers below the series of graphs indicate the cM position at the end of each linkage group. (PNG) [file pone.0210611.s013.png]

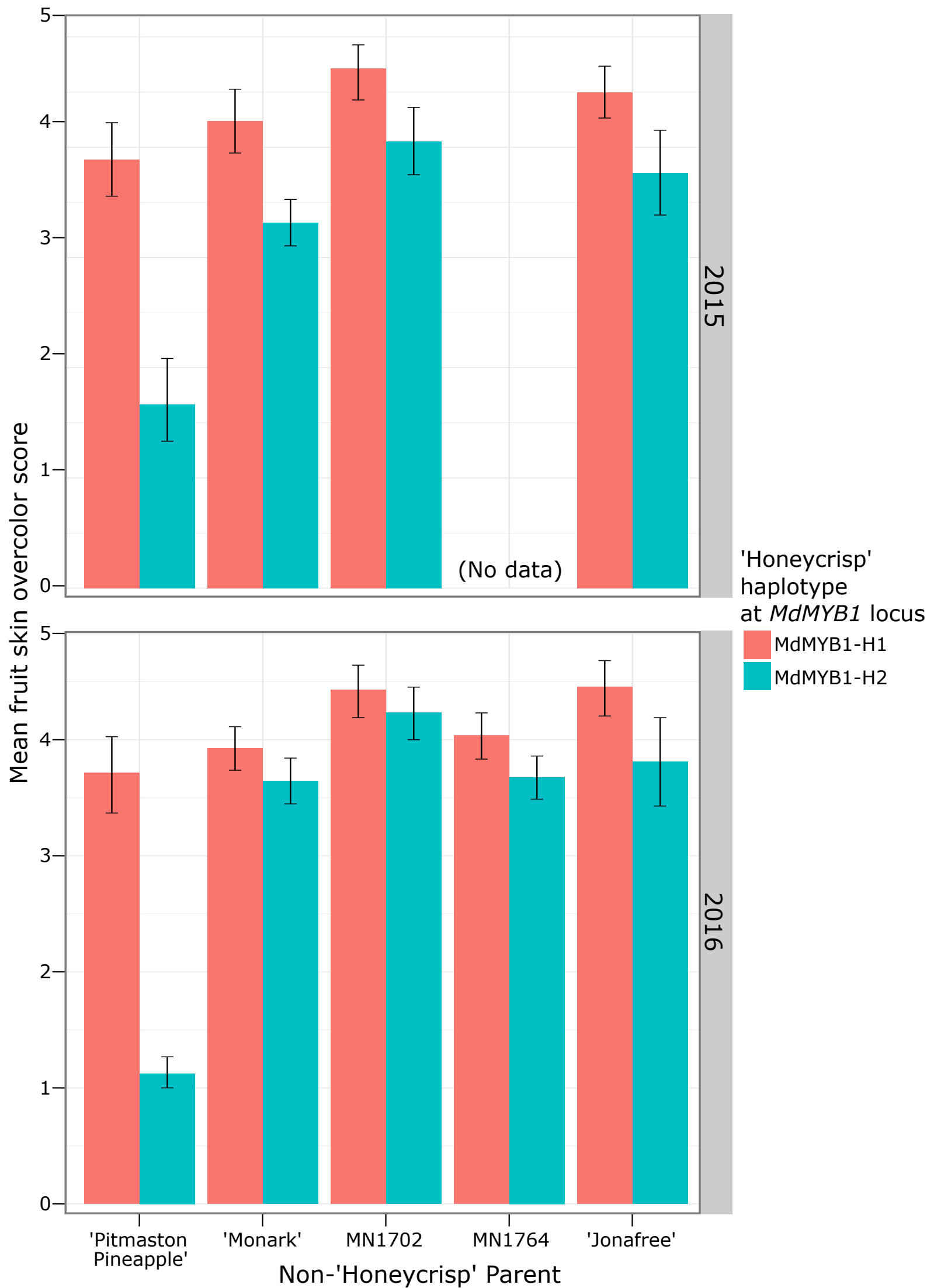

Supplement: S5 Fig — (PDF) [file pone.0210611.s014.pdf]

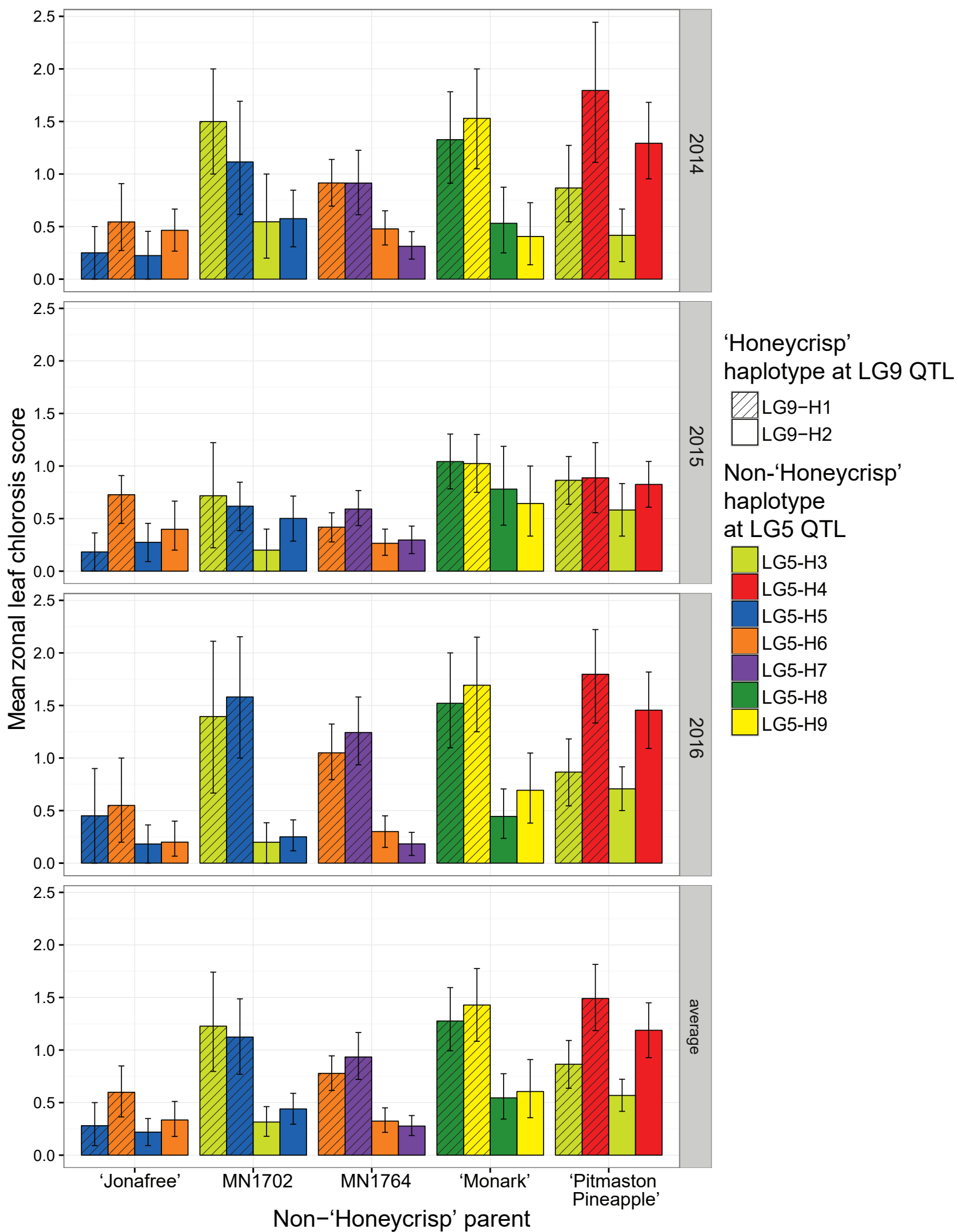

Supplement: S6 Fig — (PDF) [file pone.0210611.s015.pdf]

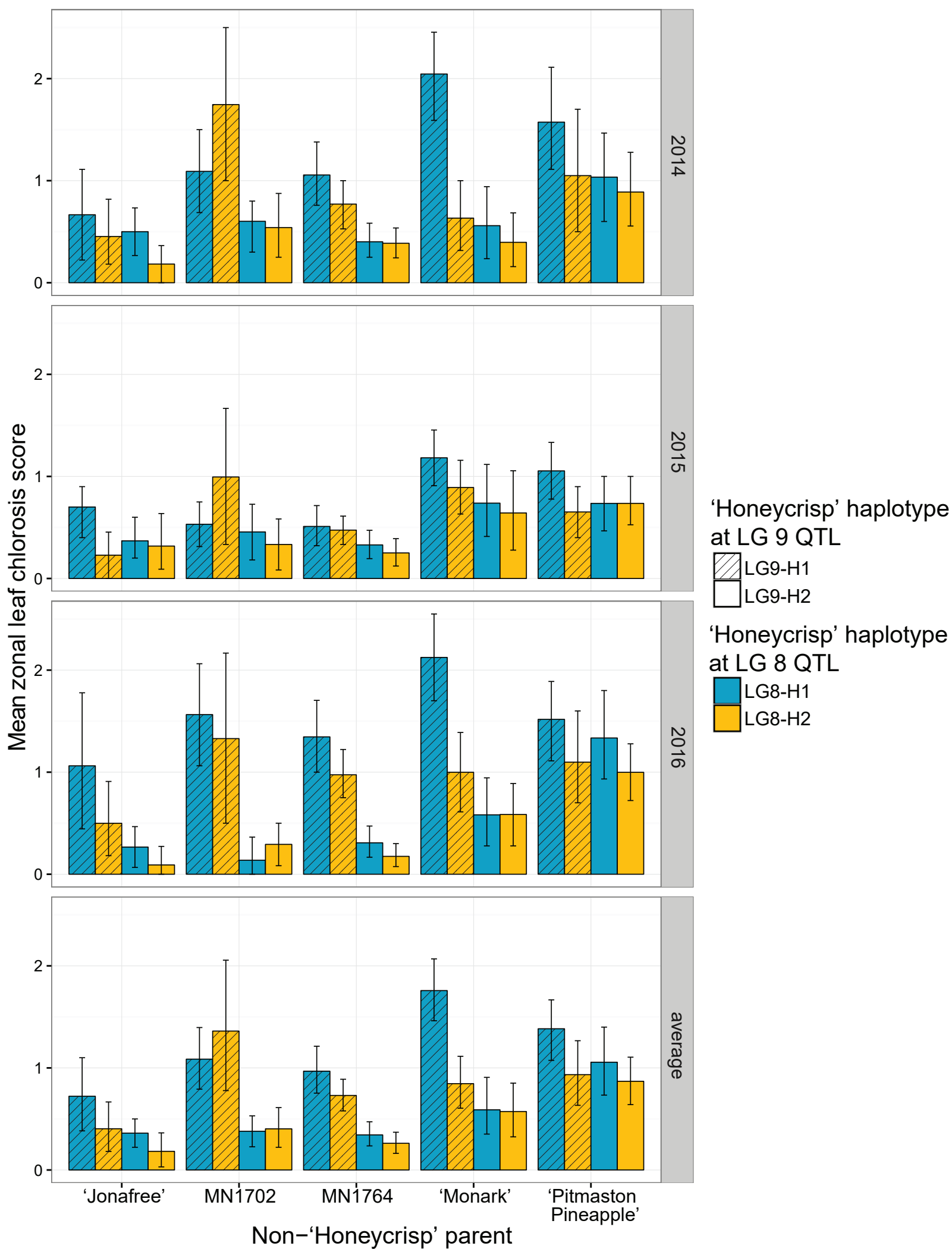

Supplement: S7 Fig — (PDF) [file pone.0210611.s016.pdf]
